# Anatomic Association of the Proximal Fingernail Matrix to the Extensor Pollicis Longus Tendon: A Morphological and Histological Study

**DOI:** 10.3390/jcm7120465

**Published:** 2018-11-22

**Authors:** Patricia Palomo-López, Ricardo Becerro-de-Bengoa-Vallejo, Daniel López-López, César Calvo-Lobo, Manuel Herrera-Lara, Jorge Alfonso Murillo-González, Marta Elena Losa-Iglesias

**Affiliations:** 1 University Center of Plasencia, Universidad de Extremadura, 10600 Plasencia, Spain; patibiom@unex.es; 2 Facultad de Enfermería, Fisioterapia y Podología, Universidad Complutense de Madrid, 28040 Madrid, Spain; ribebeva@ucm.es; 3 Research, Health and Podiatry Unit, Department of Health Sciences, Faculty of Nursing and Podiatry, Universidade da Coruña, 15403 Ferrol, Spain; 4 Nursing and Physical Therapy Department, Faculty of Health Sciences, Institute of Biomedicine (IBIOMED), Universidad de León, 24401 Ponferrada, Spain; ccall@unileon.es; 5 Department of Human Anatomy and Embryology, Faculty of Medicine, Madrid Complutense University, 28040 Madrid, Spain; manueleh@pdi.ucm.es (M.H.-L.); jmurillo@med.ucm.es (J.A.M.-G.); 6 Faculty of Health Sciences, Universidad Rey Juan Carlos, 28670 Madrid, Spain; marta.losa@urjc.es

**Keywords:** nail bed, nail matrix, extensor pollicis longus tendon, nail deformity

## Abstract

Background: Extensor tendon disorders may cause severe functional impairments, and there is a lack of knowledge about their anatomic associations with the proximal fingernail matrix. Objective: To delineate the association between the distal extensor pollicis longus tendon (EPLT) insertion and the limit of the fingernail matrix in the thumb. Methods: The limit of the fingernail matrix and the distal bony insertion of the EPLT were identified in five thumbs from fresh-frozen human cadavers. An additional five thumbs were fixed and the longitudinal thumb sections were histologically analyzed. Results: The terminal limit of the matrix and fingernail was dorsal and overlapped to the EPL tendon, which was located between the fingernail matrix and the phalanx, and extended dorsally to the distal section of the terminal phalanx in all ten thumb bodies. Conclusion: The fingernail matrix is not directly inserted into the periosteum of the dorsal section of the base to the distal phalanx, because this anatomic relationship is separated by the deep fibers of the EPLT.

## 1. Introduction

Thumb extension is carried out by the abductor pollicis longus (APL), extensor pollicis longus tendon (EPLT), and extensor pollicis brevis muscles (EPB) [1]. Injuries to the tendons of these extensor muscles are very common (61%) and may occur more commonly than flexor tendon conditions [2,3], because of their superficial location and the lack of overlying subcutaneous tissue. Extensor tendon (ET) injuries may cause a severe functional impairment [4], and these conditions may often involve the thumb tip and the functionally integrated nail matrix, plate, and bed [5,6]. 

Surgery on these areas and the distal phalanx after trauma or other conditions, including infection, tumors, iatrogenic injury, and degenerative conditions, may require a detailed understanding of the anatomy and of the association between the fingernail and its surrounding tissue. Indeed, the fingernail matrix seems to be the only source of the fingernail plate, and is particularly vulnerable to both injury and the subsequent structural alterations after the surgical display of the dorsal section of the thumb and the fingernail plate. Therefore, learning about the proximal bound of the matrix may be very important for minimizing surgery-related injuries and deformities of the thumb [7,8]. 

Numerous studies have examined the anatomy of the fingernail unit [7,8,9,10,11,12,13,14,15], but to our knowledge, there is no prior research evaluating the anatomic association between the proximal fingernail matrix and the final EPLT insertion in the thumb. In fact, there is conflicting information regarding the terminal EPLT insertion, which was previously described as being inserted both directly into the dorsal section of the distal phalange [16] and into the dorsal aponeurosis of the thumb, before continuing distally within the aponeurosis and inserting into the posterior dorsal base of the distal phalange [16]. A recent study has shown that the extensor hallucis longus tendon (EHLT) of the big toe may be located between the matrix and the phalanx, with the tendon bundles extending dorsally to the distal section of the distal phalange [17]. Thus, our objectives were to measure and evaluate the anatomy of the final EPLT and its near structures, and to ascertain whether the association between the nail matrix and the EPLT may be similar to that seen with the EHLT. 

## 2. Materials and Methods

This research was favorably approved by the Research Ethical Committee of the King Juan Carlos University, Ethical Committee Number: 0801201800818. Ten fresh frozen human cadaver thumbs, from seven males and three females, without evidence of trauma, were included in this investigation. The mean age was 76.5 ± 7.47 years (range 60 to 85 years old). The thumbs were recruited from five right and five left hands. The bodies belonged to the Scientific Anatomy Center, S.L. (Valencia, Spain).

Five thumbs were used for the histological analysis and five thumbs were used for the anatomical dissection. For the histological analysis, the tissue was dipped in a neutral formaldehyde solution for seven days. It was also decalcified with nitric acid containing a 5% concentration percentage, dehydrated, and embedded to paraffin. Then, the paraffin slabs were sliced (12- to 15-µm-thick sagittal cuts) and stained with tetrachrome V.O.F-III G.S stain (light-green SF/fast-green FCF, methyl-blue, Orange G, and fuchsine acid) [18], collagen fiber-specific picrosirius red stain [19], or via the molybdenum blue reaction [20], as previously described, with only minor modifications. A microscopic examination was used to evaluate the insertion or appearance of the EPLT according the dorsal section of the distal phalange. 

For the anatomical dissection, a lengthwise dorsal hide cut was made along the length of the middle and distal thumbs, and was extended through the eponychium. Two radial incisions were also made proximal to the junctions of the fingernail fold and fingernail walls [21,22]. The hide flaps were retracted to present the dorsal section of the matrix and the entire length of the fingernail plate. The fingernail plate was then deleted atraumatically (preserving the sections of the (1) matrix, (2) fingernail bed, and (3) eponychium) by firstly loosening it from the matrix using a fine elevator, and then gently elevating it, prior to its sharp excision, which enabled the accurate evaluation of the nail matrix borders. The proximal section of the fingernail matrix was evaluated by visual examination, and constituted the proximal section of the fingernail bed. The fingernail matrix is paler in color than the nail bed.

**Figure 1:**
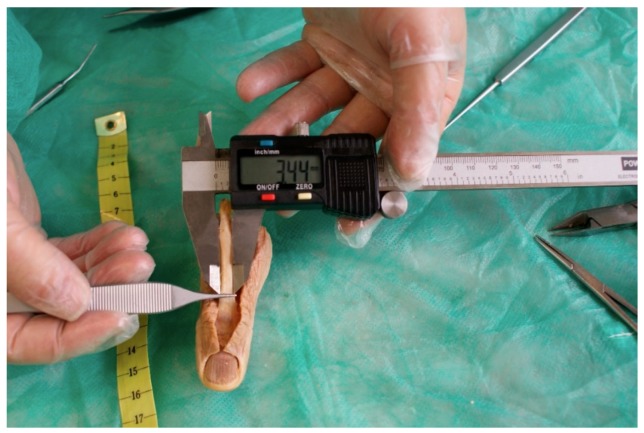
Exposing the nail matrix, the distal interphalangeal joint, and the terminal extensor pollicis longus tendon.

The proximal section of the fingernail matrix with respect to the distal limit of the final bony insertion of the EPLT was visually evaluated and confirmed using a 25× magnification optical microscope. The insertion of the final EPLT was identified by visual examination, and confirmed by verifying the section at which a thin clamp could not be passed distally beneath the tendon (Figure 1). 

**Figure 2:**
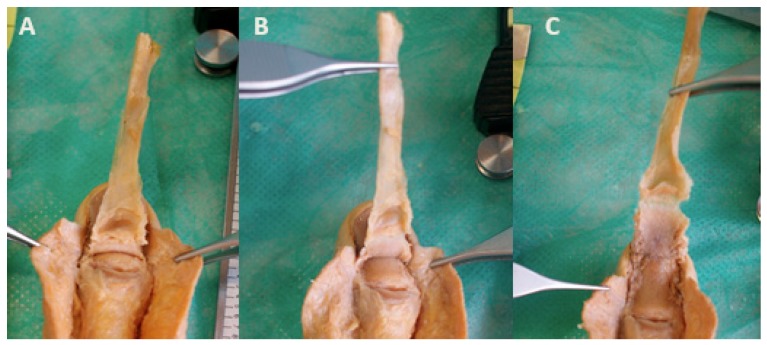
Exposing the distal insertion of the extensor pollicis longus tendon at the base of distal phalanx to the point of the proximal nail matrix fold. (**A**) The tendon attached to the dorsal aspect of the base of the distal phalanx from the distal interphalangeal joint line to the tendon reaching the proximal nail fold matrix. (**B**) The tendon at the middle aspect of the distal phalanx in the same specimen. (**C**) The tendon reaching the distal aspect of the distal phalanx.

To observe the termination point of the EPLT, it was released from the base of the terminal phalange using a no. 15 surgical blade, and was resected from the dorsal section of the distal phalanx bone until its disinsertion, as it was previously described [23] (Figure 2). Each measurement was carried out using Liquid Cristal Display (LCD) digital high precision calipers, as was previously informed [24], and with a high resolution related to a 0.01 mm in the distal inter-phalangeal joint (DIPJ) line as the reference point. The registrations were marked by placing each of the caliper tips on the proximal and distal ends of the length of the dorsal section of the terminal phalange. All of the registrations of the variables were collected as the range and mean ± standard deviation (SD).

## 3. Results

**Figure 3:**
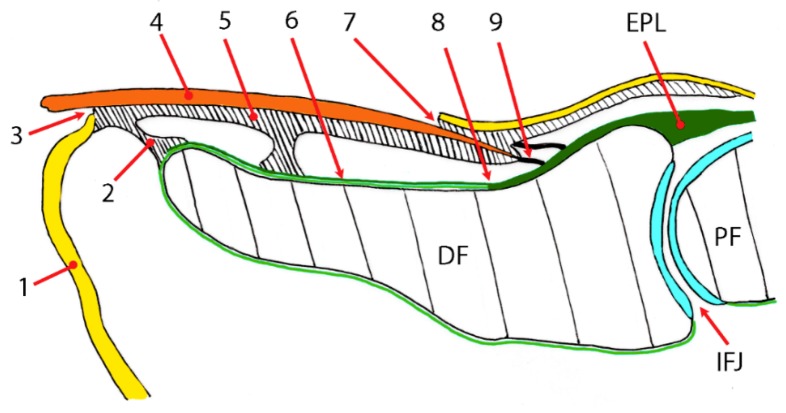
Drawing corresponding to Figure 4: (1) epidermis; (2) hyponychial phalangeal (anterior) ligament; (3) hyponychium; (4) nail plate; (5) nail matrix; (6) the superficial extensor pollicis longus (EPL) tendon bundle to the distal phalanx of the thumb aponeurotic expansion; (7) cuticle (8) superficial EPL tendon bundle that continues to the distal phalanx of the thumb; (9) superficial fibers of the EPL bundles. DF—distal phalanx; EPL—extensor pollicis longus; IFJ—interphalangeal joint; PF—proximal phalanx.

The proximal edge of the fingernail matrix and the terminal EPLT insertion were clearly identified in all of the thumb specimens. We observed that the EPLT was attached to the posterior dorsal base of the terminal phalange, and had superficial fibers that ran distally along the complete dorsal aspect of the distal phalanx until its end, where the EPLT was disinserted in all of the specimens (Figure 3). Our measurements are shown in Table 1. Thus, in all of the specimens, the EPLT reached the distal part of the distal phalanx, and covered it totally. 

## 4. Discussion

Several studies in hallux toenail digits have noted the close association between the EPLT and the fingernail matrix [8,10,11,12,13,14,15]. Indeed, Reardon et al. noted that the insertion of the ET was always the most proximal to the proximal section of the matrix [12], and another study showed that the proximal limit of the matrix in the digit was extremely close (1.2 mm separated) to the terminal ET bony insertion [25]. Nevertheless, previous studies have not evaluated the precise anatomic distance between these structures in the thumb, as we reported in this study. 

**Figure 4:**
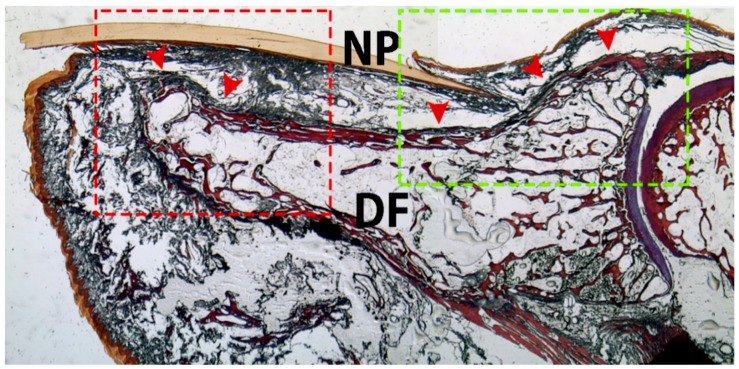
Sagital section of the thumb. The superficial fibers of the EPL tendon bundles extend to the dorsal aspect of the distal phalanx of the thumb (arrowheads). Convex and concave structures form the interphalangeal joint of thumb. The red and green squared areas correspond to Figure 5 and Figure 6, respectively. NP—nail plate; DF—distal phalanx. (5× magnification). Tetrachrome VOF-III GS stain (light green SF/or fast green FCF, methyl blue, Orange G, and acid fuchsin).

**Figure 5:**
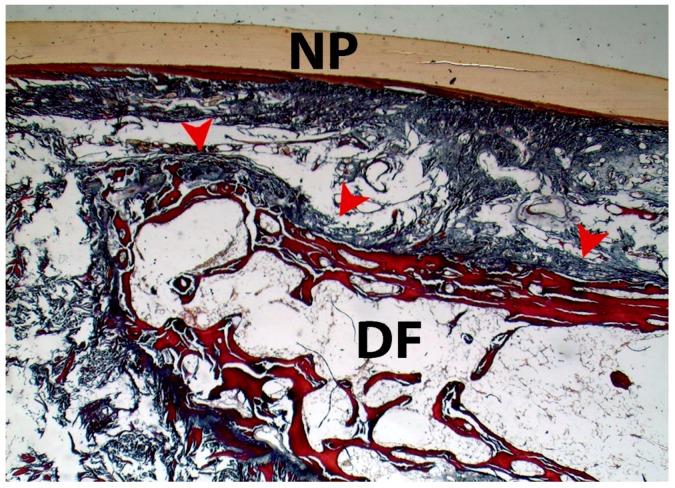
High-power magnification of the red squared area of Figure 4. The superficial EPL tendon bundle attached to the distal phalanx of the thumb (arrowheads). NP—nail plate; DF—distal phalanx. (10× magnification). Tetrachrome VOF-III GS stain (light green SF/or fast green FCF, methyl blue, Orange G, and acid fuchsin).

The current study also analyzed how the fingernail is functionally tied to the dorsal distal section of the terminal phalange and some distal interphalangeal joint structures, including the ET fibers and collateral ligaments. We show that the bony insertion of the EPLT was enveloped to the dorsal aspect of the distal phalange, and the collateral ligaments formed an integrated network on the sides of the joint, thereby helping to anchor the nail margins (Figure 4 and Figure 5). These superficial fibers of the EPLT, attached along the dorsal section of the distal phalange of the thumb, merged with the dorsal section of the distal phalange and with several cutaneous ligaments, which ensured the fatty cushion of the digit pulp to the skin (Figure 5). 

Here, the insertion of the EPLT into the dorsal posterior base of the distal phalanx is also visible, and the collagen bundles are arranged in parallel with the periosteum of the dorsal section of the distal phalange surface. The complex anchoring phenomena of the EPLT showed its principal bony attachment to the plus proximal dorsal part of the distal phalange; nevertheless, other researchers found a structure called the “deep lamina” of the ET [26,27], which blended with the outer fibrous layer of the periosteum, and another group observed that the fibers from the ET splitted and fused with a strikingly thick periosteum over the dorsal surface of the distal phalanx [28]. 

Other researchers reported that the fingernail appeared from the matrix, which seemed to be usually attached to the periosteum of the distal phalanx, and this structure firmly anchored the fingernail plate in place to the fingernail bed [29,30]. Another research group that examined the extent of the matrix in fingernails, showed that the matrix did extend to the insertion of the ET, although the distance was not quantified [10]. In the thumb, the EPLT runs longitudinally, sandwiched between the matrix (dorsal to the tendon) and distal phalanx (volar to the tendon), and envelops the phalanx after its insertion at the base. 

The EPLT continues beyond the dorsal aspect of its proximal bony attachment, and sends a deep lamina or deep ET bundles to continue with a gross periosteum, covering the dorsal distal section of the distal phalanx, as shown in Figure 4 and Figure 5.

The fingernail matrix of the thumb is not enclosed to the periosteum of the dorsal section of the radix of the distal phalange, as reported previously, because the EPLT runs deep along the nail matrix, and the tendon is dorsal to the bone and extends until the distal dorsal section of the distal phalanx. Our anatomic and histologic studies demonstrate that the proximal limit of the digit matrix and fingernail bed in the thumb are dorsal, and overlap the final EPLT until its distal bony insertion in all of the samples of the specimens.

**Figure 6:**
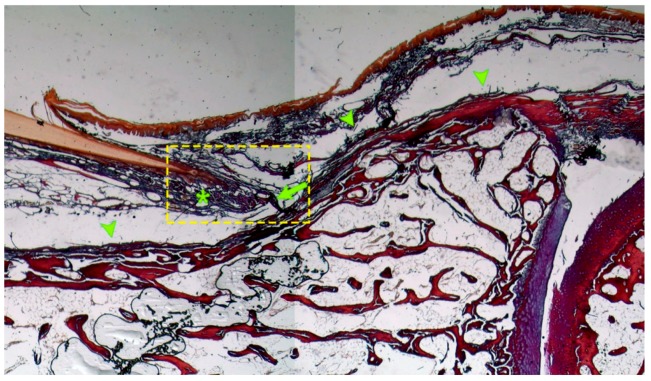
High-power magnification of the green squared area of Figure 4. Deep fibers of the extensor pollicis longus tendon (EPLT) bundles run along the dorsal aspect of the distal phalanx of the thumb (arrowheads). Superficial fibers of the EPL (arrow) are attached at the base of the ventral nail matrix (asterisk) and to the underlying bone crossing the superficial fibers of the EPLT bundles (10× magnification). Tetrachrome VOF-III GS stain (light green SF/or fast green FCF, methyl blue, Orange G, and acid fuchsin).

Surprisingly, we found that the posterior ligament that was inserted into the ventral fingernail matrix originated from the superficial fibers of the EPLT, and anchored these two structures together (Figure 6). This contradicts previous studies, which indicated that the EPLT was enclosed to the base of the ventral matrix to the underlying bone at this proximal level, a small area, and was segmented by the bony insertions called the matricophalangeal hind ligament [8,31,32].

**Figure 7:**
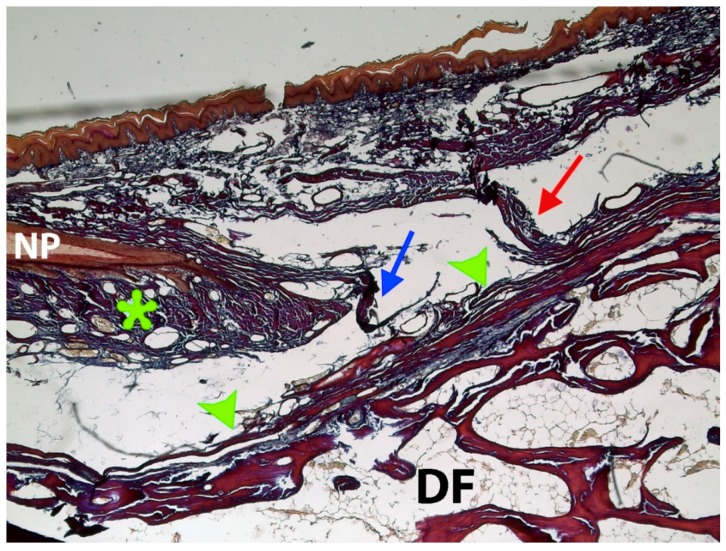
High-power magnification of the yellow squared area of Figure 6. Detail of the superficial fibers of the EPL bundles (blue and red arrows) attached at the base of ventral matrix (asterisk) to the underlying distal phalanx (DF) bone, crossing the deep fibers of the extensor pollicis longus tendon (EPLT) bundles (green arrowheads). Superficial fibers of the EPL tendon expansion to the eponychium (blue and red arrow), which run distally along the complete dorsal aspect of the distal phalanx, until its end (20× magnification). Tetrachrome VOF-III GS stain (light green SF/or fast green FCF, methyl blue, Orange G, and acid fuchsin).

Using high magnification (Figure 7), we observed that the posterior ligament is attached to the base of the ventral matrix to the underlying bone, but crosses the superficial fibers of the EPL bundles. We agree with McGonagle’s (2008) “bilaminar” description of the fibers, which extended from the EPLT with both superficial lamina and deep lamina, which formed a thick periosteum. Nevertheless, this previous study did not show that the “deep lamina” of the EPLT inserted into the plus distal aspect of the distal phalanx, as we have demonstrated [26].

Across the dissection and histologic studies, we have displayed that the EPLT of the thumb terminates in the dorsal zone of the distal phalange of the thumb, specifically in its most distal zone. There is a close interrelationship between the EPLT and the fingernail matrix, as the nail is functionally linked to the distal area of the distal phalanx and the structures of the distal interphalangeal joint, including the EPLT. These findings demonstrate that the EPLT is inserted inside the dorsal and distal zone of the distal phalange, linking with the periosteum, whereas anterior studies [33,34,35,36] reported that this tendon was enclosed to the dorsal zone of the terminal phalange base. 

The primary limitation of our study is that we could not determine whether the observed superficial lamina of the EPLT is a true bundle of this tendon, or whether it is a ligament; further research is required to clarify this. 

### Clinical Significance

The most important functions of the fingernail are proprioception related, according Sherrington, with the body segments and movement in space [37], protection of the digit, and check of the thumb pulp. Harm to the matrix may lead to important alterations of the fingernail plate [24] and the situation of the matrix related to the EPLT, which may present important clinical implications. 

Our findings improve the field’s understanding of the nail anatomy, which could be clinically useful in certain pathologies, including paronychia, tendon ruptures, extensor injuries psoriasis, arthritis, steroid injections, fractures, repetitive activities, or other systemic diseases and infections, or inflammation spread along tendons [38,39,40].

The EPLT is not inserted into the base of the distal phalange, but instead runs along its entirety. This could be relevant to surgeons, in order to avoid the intra surgery hurt of the fingernail matrix as well as post-surgery problems. Indeed, the fingernail matrix infections after a fingernail operation surgery could influence the EPLT below, spreading the risk of infection proximally, along the trajectory of the affected EPLT. 

The normal fingernail consists of the proximal nail fold, nail matrix, nail bed, and hyponychium. Beneath the nail matrix and nail bed, there is a specialized nail mesenchyme, called the onychodermis [41,42], which is associated with the attenuated subcutaneous fat. Regarding the distances from the different areas of the nail unit to the phalangeal bone, it is the shortest distance from the nail matrix area [43]. Other authors stated that the nail bed was directly attached to the dorsal surface of the distal phalanx of the thumb [44].

## 5. Conclusions

We found that the EPLT is localized between the fingernail matrix and the phalange, and that the tendon’s superficial fibers extend dorsally to the distal area of the distal phalange. We demonstrate that the thumb’s fingernail matrix is not enclosed to the periosteum of the dorsal area of the base of distal phalange, as it was previously reported, because the deep lamina of the EPLT is directly below the fingernail matrix and dorsal to the bone. Our anatomic and histologic studies demonstrate that the proximal limit of the matrix and fingernail bed of the human digit are dorsal, and overlap the terminal EPLT until its distal osseous insertion of the distal phalanx in all of our specimens, and that the superficial fibers arise from the EPLT to the ventral nail matrix. This work contributes additional knowledge to what is already known about the anatomy of the thumb, which is important in the relevant surgical procedures, in order to improve outcomes and reduce complications. 

## Figures and Tables

**Table 1:** Measurements of the length of the dorsal aspect of the distal phalanx of the thumb, until its end, where the extensor pollicis longus (EPL) tendon is disinserted. SD—standard deviation.

	Specimen Number					Total (*N* = 5)
	1	2	3	4	5	Total Mean ± SD (range)
Length (mm)	8.80	11.20	12.56	10.35	11.89	10.96 ± 1.46 (8.80–12.56)
